# Automated Non-Sterile Pharmacy Compounding: A Multi-Site Study in European Hospital and Community Pharmacies with Pediatric Immediate Release Propranolol Hydrochloride Tablets

**DOI:** 10.3390/pharmaceutics16050678

**Published:** 2024-05-17

**Authors:** Niklas Sandler Topelius, Farnaz Shokraneh, Mahsa Bahman, Julius Lahtinen, Niko Hassinen, Sari Airaksinen, Soumya Verma, Ludmila Hrizanovska, Jana Lass, Urve Paaver, Janika Tähnas, Catharina Kern, Frederic Lagarce, Dominic Fenske, Julia Malik, Holger Scherliess, Sara P. Cruz, Mattias Paulsson, Jan Dekker, Katja Kammonen, Maria Rautamo, Hendrik Lück, Antoine Pierrot, Stephanie Stareprawo, Marija Tubic-Grozdanis, Stefanie Zibolka, Uli Lösch, Martina Jeske, Ulrich Griesser, Karin Hummer, Andreas Thalmeier, Anna Harjans, Alexander Kruse, Ralph Heimke-Brinck, Karim Khoukh, Fabien Bruno

**Affiliations:** 1 CurifyLabs Oy, Salmisaarenaukio 1, 00180 Helsinki, Finland; farnaz.shokraneh@curifylabs.com (F.S.); julius.lahtinen@curifylabs.com (J.L.); soumya.verma@curifylabs.com (S.V.);; 2 Pharmaceutical Sciences Laboratory, Åbo Akademi University, Artillerigatan 6A, 02520 Turku, Finland; 3 Tartu University Hospital, 50406 Tartu, Estonia; jana.lass@kliinikum.ee; 4 Institute of Pharmacy, Tartu University, 50411 Tartu, Estonia; urve.paaver@ut.ee; 5 Tartu Raekoja Apoteek, 51003 Tartu, Estonia; 6 Universitätsklinikum Münster, 48149 Münster, Germany; 7 CHU d’Angers, 49100 Angers, France; frederic.lagarce@univ-angers.fr; 8 Helios Klinikum Erfurt, 99089 Erfurt, Germany; dominic.fenske@helios-gesundheit.de; 9 Asklepios Klinik Nord, 22417 Hamburg, Germany; j.malik@asklepios.com; 10 Kiel Apotheke am Bebelplatz, 24146 Kiel, Germany; scherliess@bebelplatz-apotheke.de; 11 Hietalahden Apteekki, 65130 Vaasa, Finland; vaasan.8.apteekki@apteekit.net; 12 Department of Women’s and Children’s Health, Uppsala University, Akademiska Sjukhuset, SE-751 85 Uppsala, Sweden; 13 UMC Utrecht, 3584 CX Utrecht, The Netherlands; 14 University Pharmacy, 00100 Helsinki, Finland; katja.kammonen@ya.fi; 15 HUS Helsinki University Hospital, 00029 Helsinki, Finland; maria.rautamo@hus.fi; 16 Faculty of Pharmacy, University of Helsinki, 00100 Helsinki, Finland; 17 UKSH Universitätsklinikum Schleswig-Holstein, 24105 Kiel, Germany; hendrik.lueck@uksh.de; 18 UKSH Universitätsklinikum Schleswig-Holstein, 24105 Lubeck, Germany; 19 Centre Hospitalier Universitaire Vaudois, 1005 Lausanne, Switzerland; 20 Universitätsklinikum Halle (Saale), 06120 Halle (Saale), Germany; stephanie.stareprawo@uk-halle.de; 21 Universitätsmedizin der Johannes Gutenberg-Universität Mainz, 55131 Mainz, Germany; apo-studi@unimedizin-mainz.de; 22 Universitätsklinikum Magdeburg A.ö.R., 39120 Magdeburg, Germany; stefanie.zibolka@med.ovgu.de; 23 Universitätsspital Basel, 4031 Basel, Switzerland; uli.loesch@usb.ch; 24 Tirol Kliniken Innsbruck, 6020 Innsbruck, Austria; 25 Institute of Pharmacy, University of Innsbruck, 6020 Innsbruck, Austria; ulrich.griesser@uibk.ac.at; 26 Landeskrankenanstalten-Betriebsgesellschaft—KABEG (Klagenfurt), 9020 Klagenfurt am Wörthersee, Austria; 27 Ludwig-Maximilians-Universitäts Klinikum, 81377 München, Germany; andreas.thalmeier@med.uni-muenchen.de; 28 Universitätsklinikum Heidelberg, 69120 Heidelberg, Germany; 29 Charité Universitätsmedizin Berlin, 10117 Berlin, Germany; alexander.kruse@charite.de; 30 University Hospital Erlangen (Apotheke des Universitätsklinikums Erlangen), 91054 Erlangen, Germany; ralph.heimke-brinck@uk-erlangen.de; 31 Delpech Pharmacy, 75006 Paris, Francefabienbruno@delpechparis.com (F.B.)

**Keywords:** automated compounding, hospital and community pharmacies, pharma inks, integrated quality control

## Abstract

Pharmacy compounding, the art and science of preparing customized medications to meet individual patient needs, is on the verge of transformation. Traditional methods of compounding often involve manual and time-consuming processes, presenting challenges in terms of consistency, dosage accuracy, quality control, contamination, and scalability. However, the emergence of cutting-edge technologies has paved a way for a new era for pharmacy compounding, promising to redefine the way medications are prepared and delivered as pharmacy-tailored personalized medicines. In this multi-site study, more than 30 hospitals and community pharmacies from eight countries in Europe utilized a novel automated dosing approach inspired by 3D printing for the compounding of non-sterile propranolol hydrochloride tablets. CuraBlend^®^ excipient base, a GMP-manufactured excipient base (pharma-ink) intended for automated compounding applications, was used. A standardized study protocol to test the automated dosing of tablets with variable weights was performed in all participating pharmacies in four different iterative phases. Integrated quality control was performed with an in-process scale and NIR spectroscopy supported by HPLC content uniformity measurements. In total, 6088 propranolol tablets were produced at different locations during this study. It was shown that the dosing accuracy of the process increased from about 90% to 100% from Phase 1 to Phase 4 by making improvements to the formulation and the hardware solutions. The results indicate that through this automated and quality controlled compounding approach, extemporaneous pharmacy manufacturing can take a giant leap forward towards automation and digital manufacture of dosage forms in hospital pharmacies and compounding pharmacies.

## 1. Introduction

The field of pharmacy compounding, where personalized dosage forms are manufactured when there are no suitable treatments available as market-authorized drugs, is witnessing a transformative evolution with advancements inspired by cutting-edge technologies reshaping the landscape of pharmaceutical manufacturing. In this context, automated dosing technologies, inspired by the principles of 3D printing and other technologies, have emerged with the potential to redefine pharmacy compounding [1,2]. Dosing automation offers a solution into the future of pharmaceutical production, promising greater precision, efficiency, and accessibility in the creation of customized medications and standardizing workflows.

Additive manufacturing, commonly referred to as three-dimensional (3D) printing (3DP), entails the fabrication of customized 3D structures utilizing digital computer-aided design (CAD) files, employing a layer-by-layer approach [3,4,5]. Within the realm of pharmaceuticals, 3DP, and other printing technologies, has already increasingly received substantial attention for its capacity to generate individualized and unique medications or 3D-printed tablets since 2011 [6,7,8,9,10]. Pharmaceutical 3DP allows for the creation of medications with a wide spectrum of dosage levels, shapes, flavors, colors, drug combinations, and drug release profiles, all tailored to the specific needs of each patient or disease condition [11,12,13,14]. It has been stated that these attributes make this technology particularly advantageous for diverse patient groups, including pediatrics, geriatrics, those on multiple medications, and individuals with rare diseases. There are many printing technologies, but semi-solid extrusion (SSE) 3D printing has exhibited significant promise for integration within a clinical setting due to the possibility use low temperatures and its relative non-complexity over other 3DP techniques. As an example, the manufacture of orodispersible films by 2D or 3D printing has been reported to be successful for on-demand manufacturing of patient-specific doses [15,16]. 

However, even though 3DP has emerged as a proposed alternative to conventional pharmaceutical compounding, enabling point of care treatments, it has evident drawbacks, as layer-by-layer deposition is often very time-consuming and does not lend itself to the manufacture of conventional pharmacy-tailored products where simplicity, speed, and time-savings are of essence. The main challenge with 3D printers is their lack of practicality in high-demand settings. They often operate at a slower pace compared to the needs of a busy pharmacy, where speed and efficiency are paramount [17,18] Additionally, the complexity of these printers can make them less user-friendly, requiring specialized training and expertise, which may not be feasible in most pharmacy settings [8]. Therefore, alternative practical, easy-to-use automation approaches that hold the potential to mitigate dosing errors and contamination, enhance product quality, and streamline the compounding process through automation, replacing manual methods and ultimately contributing to improved patient care, are required. 

**Figure 1:**
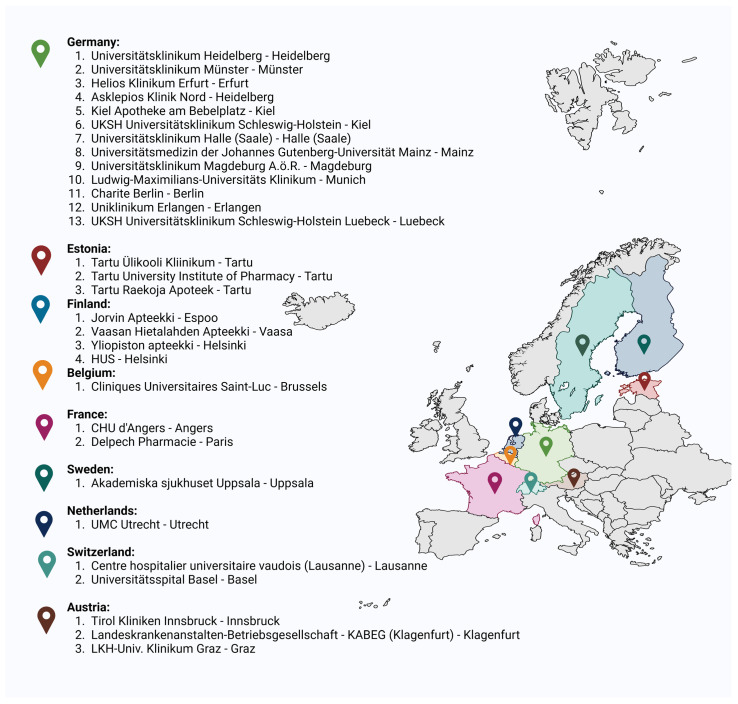
European hospitals and community pharmacies that took part in this study.

In this unique multi-site study, conducted in a total of 30 hospital and community pharmacies in eight different countries in Europe, the capabilities of the introduced non-sterile automated dosing technology were explored (Figure 1). This collaborative effort, known as the “alpha tests”, aimed to investigate the performance of the technology in manufacturing propranolol hydrochloride tablets specifically tailored for pediatric use. The alpha tests primarily focused on evaluating dosing accuracy, including both mass and content uniformity, to ensure demonstration of in-process control of the tablets produced. Secondary aspects were the evaluation of user-friendliness and ease of use of the system in a pharmacy setting.

The alpha tests were executed in four distinct stages, with each stage building upon the knowledge gained from the previous one. The technology and a gel-based formulation underwent iterative enhancements and refinements after each stage, guided by valuable feedback from the participating pharmacists and end-users. One goal of this study was to develop and produce propranolol hydrochloride tablets that meet the pharmacopeial quality standards, but also address the unique needs and requirements of pediatric patients. Currently, there is no suitable marketed propranolol formulation available for administration for children.

The aim of this study was to develop an automated extrusion-based material deposition technology to be used with GMP-manufactured excipient bases to offer a more viable alternative for rapid automated compounding without the restrictions related to speed and user-friendliness of most 3D printing technologies. This article presents a comprehensive overview of the alpha tests, showcasing the journey from concept to realization and the collaborative efforts of experts in the pharmaceutical field. It aims to highlight the role played by automated dosing technology, emphasizing its potential to revolutionize the way we approach pharmacy compounding. 

## 2. Materials

The excipient base (pharma-ink) for the automated dosing process were formulated using propranolol hydrochloride (Caesar & Lorez GmbH, Hilden, Germany), polysorbate 80 (Caesar & Lorez GmbH, Hilden, Germany), and CuraBlend^®^ (CurifyLabs Oy, Helsinki, Finland). High-performance liquid chromatography (HPLC)-grade acetonitrile and methanol were sourced from Fisher Scientific (Loughborough, UK), and potassium phosphate dibasic was obtained from Sigma Aldrich (Steinheim, Germany). All chemicals and reagents were of analytical grade.

For blister packaging, 3/16″ Mini Medi-Cap^®^ Plus™ Blisters (MD425, MediDose Group, Ivyland, PA, USA) were chosen. The blister lids employed were LaserLabel™ “25” Lid-Label^®^ Cover Sheets from MediDose.com. Cartridges used in the dosing process in Phases 1 and 2 were supplied by Natural Machines, Barcelona, Spain. For Phases 3 and 4, sterilized single-use PVC syringes, 100 mL, equipped with a Luer-lock mechanism, were procured from Yangzhou Bessent Textile Trading Co., Ltd., Shanghai, China. A silicon mat was sourced from Fengyang Jiasong New Material Technology Co., Ltd., Chuzhou, China.

## 3. Methods

### 3.1. Testing Approach

**Figure 2:**
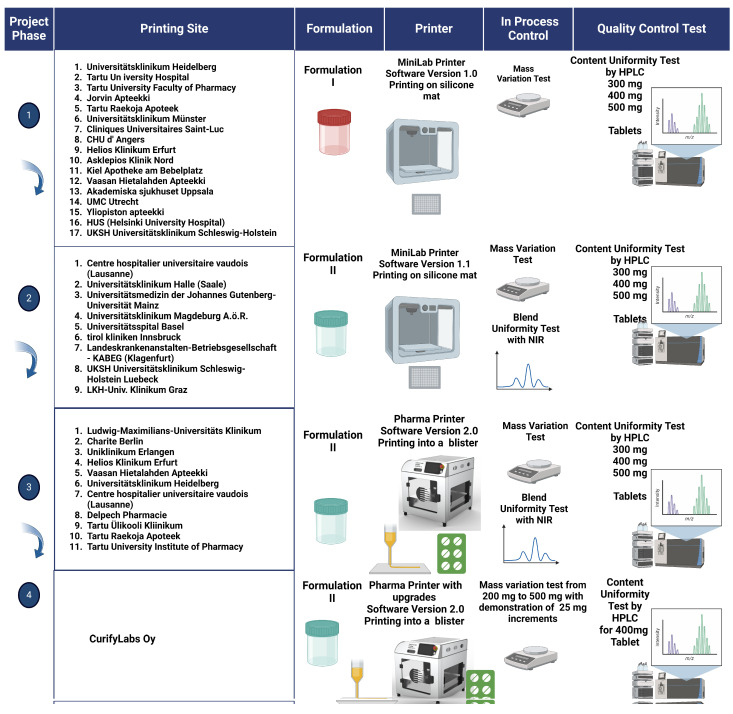
Graphical presentation of the sequential testing and the four phases of this study.

The development of the project unfolded through four phases, each designed to enhance and refine the introduced automated dosing technology for pharmacy tailored manufacturing of dosage forms. In each phase, the participants used the technology to dose several different tablets with different weights following a strict study protocol. At each participating site, the same study protocol was followed, which is explained more in detail below and graphically in Figure 2. 

**Phase 1** introduced the foundational elements: the MiniLab printer equipped with Control Software 1.0, establishing the basis for testing the automated dosing process and an initial Formulation 1.0. Key quality control features included an in-process scale for ensuring mass uniformity and high-performance liquid chromatography (HPLC) for assessing content uniformity and the use of a silicone mat for collecting the dosed tablets. Tablets were deposited on a silicone mat.

**Phase 2** built upon this initial foundation by implementing upgrades in software and enhancing the control software to version 1.1, improving the formulation with the intention to improve the precision of material blending and the blend uniformity. The phase also marked the inclusion of near-infrared (NIR) spectroscopy for measuring blend uniformity, further enhancing the project’s ability to ensure consistent product quality. Tablets were again deposited on a silicone mat.

**Phase 3** marked a significant shift towards more sophisticated technology by the introduction of the Pharma Printer, which featured advanced automated dosing technology for precise control over drug dosage, which was based on insights learned in the previous phases. This phase continued the use of the improved Formulation 2.0 and introduced Control Software 2.0, focusing on enhancing automation and process control. Quality control measures from the previous phases were maintained, and the project evolved by moving to deposit directly into blisters, streamlining the manufacturing and packaging process.

**In Phase 4**, the project continued to refine the automated dosing technology for pharmaceutical manufacturing, focusing on enhancements to the Pharma Printer. This phase introduced hardware improvements aimed at increasing the precision and efficiency of the dosing process. The use of an in-process scale for mass uniformity remained crucial in this phase, ensuring the accuracy of each dose. A significant achievement was the demonstration of dosing capabilities with weight increments of 25 mg, offering a finer level of control over medication dosing, which is vital for personalized medicine applications.

### 3.2. Preparation of the Formulation 

In the formulation process, two distinct formulations, Formulation 1.0 (Form I) and Formulation 2.0 (Form II), were prepared to investigate their impact on the deposition accuracy and content uniformity of pharmaceutical tablets. All ingredients in the formulations are suitable for pediatric use.

**Formulation I (Form I)**

The printed Form I consisted of 99% *w*/*w* gelatin-based CuraBlend^®^ as a basic formulation, and 1% *w*/*w* propranolol hydrochloride (Prop HCl) as an active pharmaceutical ingredient (API). CuraBlend^®^ is a GMP-manufactured suspending base (pharma-ink) for pharmacy compounding. CuraBlend^®^ was melted in a water bath at +45 °C (temperature range: ±3 °C) for 30 min until it reached a liquid state. Propranolol hydrochloride (API) was weighed in a metal mortar and warm CuraBlend^®^ was added to the mortar in small quantities while mixing. The resulting Formulation I was mixed thoroughly for 3–4 min. The covered mortar was then placed in a warm water bath for 5 min and mixed thoroughly again to ensure uniform mixing of the API. 

The warm Form I was poured into the metal cartridge of printer. All the air was pushed out of the cartridge until the first drops of Form I came out from the nozzle (nozzle size 15 from Natural Machines). The metal cartridge was placed inside the printer’s cartridge holder and left to sit for 15 min at +42 °C before starting the print. 

The formulations were prepared at CurifyLabs 2–7 days before the actual tests at the participating pharmacies took place. The formulations were stored and transported at room temperature.

**Formulation II (Form II)**

The printed Form II consisted of 98% *w*/*w* gelatin-based CuraBlend^®^ as a basic formulation, 1% *w*/*w* polysorbate 80 (PS80) as a surfactant, and 1% *w*/*w* propranolol hydrochloride (Prop HCl) as an active pharmaceutical ingredient (API). Polysorbates (PSs) are ubiquitous in biotherapeutic formulations, and are generally considered to be safe within the ranges that are used in biotherapeutics [19].

CuraBlend^®^ was melted in a water bath at +45 °C (temperature range: ±3 °C) for 30 min until it was no longer solid in form. Prop HCL and PS 80 were weighed in a metal mortar and mixed until they formed a white paste. Warm CuraBlend^®^ was added to the mortar in small quantities while mixing. Form II was mixed thoroughly for 3–4 min. The covered mortar was then placed in a warm water bath for 5 min and mixed thoroughly again to ensure uniform mixing of the API. 

The warm Form II was transferred into a disposable syringe (100 mL) for the Pharma Printer in Phase 3 and the excess air was pushed out of the syringe until the first drops of Form II came out from the nozzle. The syringe was closed with a cap, placed inside the cartridge holder, and left to sit for 15 min at +41 °C before starting the deposition. 

It is advisable to keep the freshly prepared formulation undisturbed and tightly covered in the water bath (+42–45 °C) for 10–15 min before printing. This is to remove the air bubbles generated during the mixing and to ensure a proper deposition result.

Again, the formulations were prepared at CurifyLabs 2–7 days before the actual tests at the participating pharmacies took place. The formulations were stored and transported at room temperature.

### 3.3. Automatic Dosing Technologies and the Deposition Process

Throughout this study, the doses of propranolol HCl incorporated into the deposited tablets were carefully determined. In Formulations I and II, where the content of API in the printing mass was maintained at 1%, the doses of propranolol HCl per tablet were specified as follows: 2 mg for 200 mg tablets, 3 mg for 300 mg tablets, 4 mg for 400 mg tablets, and 5 mg for 500 mg tablets.

#### 3.3.1. MiniLab Printer and the Automated Dosing Process

An analytical balance (OHAUS Portable Precision Balance STX223 EU) was integrated into the MiniLab (Foodini, Natural Machines (NM), Spain) printer, which was controlled using an Apple iPad. The dosing process was controlled using a MiniLab tablet app installed on the Apple iPad. An order for each dosing order is created in the software where the requested tablet size, number of tablets, and printing blueprint can be selected. The parameters used for the deposition of tablets are designated by the blueprint used, which is tested and validated by CurifyLabs. The software creates a unique dosing order ID code for each order. The deposition process is then initiated from the MiniLab tablet app by selecting the required dosage order and following the instructions in the app.

The MiniLab alpha test protocol (Phase 1 and Phase 2) consisted of 9 depositions of 16 tablets, 3 parallel depositions of each tablet size, 300, 400, and 500 mg. A total of 144 tablets were dosed in each NM printer alpha test.

To prepare the formulation for the dosing, the melted propranolol formulation was poured into a metal cartridge, which was then inserted into the cartridge holder. The filled cartridge was allowed to sit for 15 min to ensure that the temperature of the formulation remained constant within the cartridge. The tablets were then deposited onto a silicone mat placed on top of the analytical balance. The tablets printed with the NM printer were kept in the refrigerator for 5 min after printing and packed into the blisters within minutes.

The integrated analytical balance recorded the weight of each individual printed tablet in milligrams, and they were sent to the MiniLab tablet app and displayed on the screen of the tablet during printing. The positions displayed on the screen correspond to the positions of the printed tablets. The software indicates if any tablets do not comply with the *Ph. Eur.* requirement of ±5% of the target weight. After the printing had finished, the weights were sent from the tablet app to the MiniLab software 1.0 and 1.1. Individual tablet weights for each print can be viewed in the software. Discrepancy between the target weight and the measured weight is also displayed in the software in milligrams and relative error percentages. All the information from the prints is stored in the software and a history of all completed prints can be viewed there.

When moving from Phase 1 to Phase 2 of the testing, improvements were made to how the information from the scale was conveyed to the tablet app. This resulted in a drop in the number of errors in the tablet weights being recorded.

#### 3.3.2. Pharma Printer and the Automated Dosing Process 

**Figure 3:**
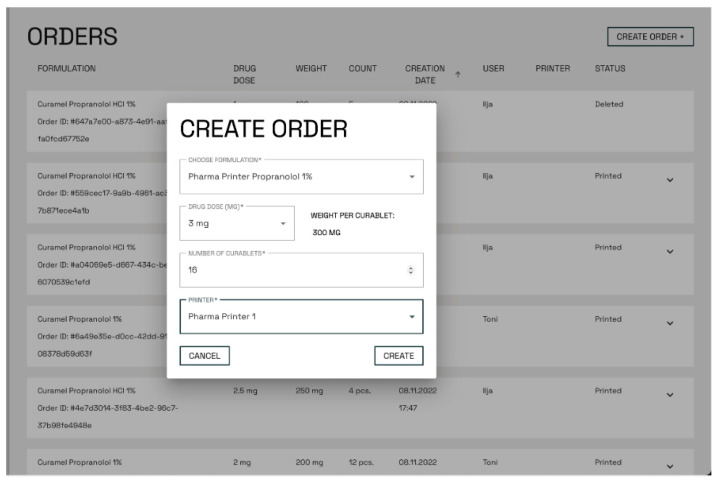
A print order is created by selecting the wanted formulation blueprint, drug dose, and number of tablets in the software.

The Pharma Printer (CurifyLabs, Finland) was developed based on user feedback and in-use experiences from the earlier alpha test phases with the NM printer. The Pharma Printer was integrated with an analytical balance (Kern PES-620-3M, Balingen, Germany) and the new CurifyLabs Control Software 2.0. Orders were created from Pharma Kit Software 2.0, where the customers chose the formulation blueprint, dose, tablet amount, and device to be used and then executed the order (Figure 3) Correct dosing parameters were tested and validated by CurifyLabs into a blueprint, which was integrated into the software. The Software creates a unique print order ID code for each order. Once the order was created, the dosing process was controlled from the printer screen, where the CurifyLabs Control Software 2.0 guided the user through the dosing process.

The Pharma Printer alpha test protocol consisted of 12 prints of 16 tablets, three depositions of each tablet size, 200, 300, 400, and 500 mg, respectively. A smaller 200 mg tablet was added to the Pharma Printer alpha test protocol (Phase 3) to test the Pharma Printer dosing accuracy with smaller tablets. A total of 192 tablets were dosed in each Pharma Printer alpha test protocol at one participating site. 

**Figure 4:**
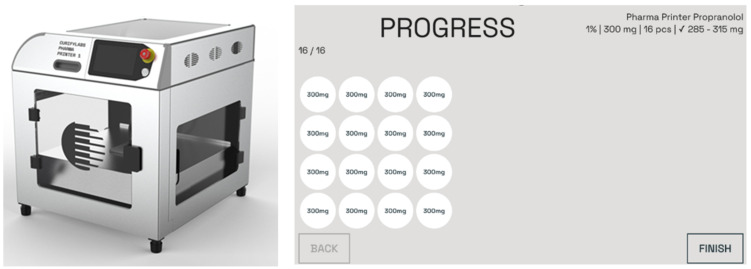
The Pharma Printer (**left**); the Pharma Printer software 2.0 (**right**) displays the deposition process, acceptance limits, and the order information during printing.

To prepare the formulation, a warm propranolol 1% formulation was poured into a single-use syringe; excess air was pushed out, the nozzle was closed with a cap, and the syringe was placed into the Pharma Printer for preheating. The heating time was 15 min, or it could be left out if the formulation was dosed earlier and was at the correct deposition temperature or preheated. Tablets were deposited directly into the blister on top of the analytical balance, and the analytical balance automatically measured and recorded each tablet’s weight twice. This update in the weighing process ensured that no extra material ended up in the tablet after the ink extrusion. The weight of each tablet was shown on the printer screen with the printing order information (Figure 4). The software used a deviation limit of ±5% for tablets over 250 mg and ±7.5% for under 250 mg according to *Ph. Eur.* uniformity of mass of single-dose preparations and indicated in red if any tablets did not comply with the *Ph. Eur.* specifications. Tablets in the blister were sealed with the blister lid. 

After the deposition was finished, dosing information was sent automatically from the printer to the Pharma Kit Software 2.0 program. Each item of deposition information can be viewed and exported from the software, including tablet weights, the discrepancy between the target and measured weight in milligrams, and relative error percentages. All the information from the tablets is stored in the software, and a history of all completed doses can be viewed and exported from there.

##### Dosing with 25 mg Increments

In the last phase, the Pharma Printer was employed with a more rigid syringe holder, and the control software was updated to feature dosing in 25 mg weight increments. The dosing was demonstrated with 200, 300, 400, 500, 225, 275, and 425 mg target weights, respectively. The testing protocol for these consisted of 21 rounds of dosing of 16 tablets, three depositions of each size. A total of 336 tablets were dosed in the Phase 4 of this study.

### 3.4. Quality Control Analysis 

#### 3.4.1. Blend Uniformity Testing with NIR Spectroscopy

A handheld NIR spectrophotometer (MicorNIR, Viavi Solutions, Santa Rosa, CA, USA) was used to collect spectra from samples of the formulations to assess the blend homogeneity of the pharma-ink API mixes. 

Formulations of Phases 1, 2, and 3 were utilized for blend uniformity testing in pharmacies before initiating the printing process. For each formulation jar, three samples were taken and analyzed by NIR spectroscopy after thorough manual mixing.

**Calibration sample preparation:** Bulk placebo CuraBlend^®^ was employed to generate a range of blends with propranolol HCl concentrations ranging from 80% to 120% *w*/*w*.

**Spectral acquisition for blend uniformity model building:** Offline spectra were acquired using the VIAVI micro NIR spectrophotometer (MicorNIR, Viavi Solutions, USA). This device utilizes a linear variable filter (LVF) and an uncooled 128-pixel InGaAs linear diode array. Spectra were collected within a spectral range of 980–1800 in reflectance mode at 0.1 ms intervals, with a 100-scan count. Offline spectra were obtained in triplicate for each sample.

**Quantitative calibration model building:** Partial least squares (PLS) regression was employed to construct the quantitative calibration model. Various PLS models were developed, incorporating diverse spectral datasets, spectral regions, and pre-processing techniques. The performance of the PLS models was assessed using parameters such as the regression coefficient (R^2^) and root mean square error (RMSE). The final PLS model was applied to the blend uniformity test of propranolol HCl pharmaceutical ink.

**Measurement process using NIRLAB app:** The NIRLAB app served as the measurement platform paired with the micro-NIR spectrophotometer. Following sample mixing, a drop of the sample was placed on the droplet collar using a disposable dropper. The middle glass lid of the droplet accessory was then closed to create a thin, uniform layer of the sample on the measuring collar.

This comprehensive methodological approach ensures accurate analysis of NIR spectra to achieve blend uniformity, facilitating robust and reliable results in our study.

**Blend Uniformity Analysis:** The NIR spectra were analyzed to ensure blend uniformity. The % blend uniformity refers to the consistency of propranolol HCl concentrations within the formulated blends compared to the predetermined range (80% to 120% *w*/*w*). This analysis provides insight into the homogeneity of the blends and verifies the accuracy of the formulation process.

#### 3.4.2. Assay and Content Uniformity Tests

A Waters AQUITY ARC High-Performance Liquid Chromatography (HPLC) system (Massachusetts, USA) equipped with a Quaternary solvent manager-R (ACQ-rQSM), a degasser, autosampler (ACQ-rFTN), and photodiode-array detector (2998 PDA) was used. The data were acquired via Empower Workstation data acquisition software. A C_18_ (2.5 µm particle size and 4.6 × 100 mm) column (Waters, Wilmslow, UK) was used. The mobile phase consisted of a mixture of 10 mM potassium phosphate dibasic buffer (pH 3.5) and acetonitrile used at flow rate of 1 mL/min. Mobile phase was filtered through a 0.2 mm membrane filter before use. The injection volume was 2 μL and UV detection wavelength was set at 230 nm. HPLC analysis was performed at gradient profile at 40 °C. Table 1 shows HPLC gradient conditions for propranolol HCl analysis.

##### Standard Preparation

Stock solutions of propranolol HCL were prepared in MilliQ water at concentrations of 500 ppm (25 mg propranolol HCL dissolved in 50 mL distilled water). The stock solution was sonicated until a clear solution was obtained. Vortexing was performed to obtain a homogenous mixture and the solution was kept in a refrigerator. A working standard solution was prepared by taking an aliquot of 1 mL stock solution in a 10 mL volumetric flask and further diluted in distilled water yielding a 50 ppm solution.

##### Tablet Sample Preparation

For tablets containing 200 mg, a 25 mL volumetric flask was used; for those containing 300 mg, a 50 mL flask was used; for tablets with a weight of 400 mg, a 50 mL flask was employed; and for tablets weighing 500 mg, a 100 mL flask was used. The tablets were placed in MilliQ water in their respective flasks and heated in a water bath at 50 °C until complete dissolution was achieved. Subsequently, the solution was allowed to cool to room temperature and then vortexed to ensure uniformity. Following this, the solution underwent filtration using disposable syringe filters with a pore size of 0.25 µm to obtain a clear solution. Finally, the filtered samples were transferred to HPLC vials for further analysis.

The acceptance value (AV) was calculated according to the procedure described in *Ph. Eur.* chapter 2.9.40, “Uniformity of dosage units”, derived from the average of specified content limits in the relevant monograph, which reflects the arithmetic mean of uniformity results across unit doses. This calculation, incorporating the acceptability constant (coverage factor) and the standard deviation of the samples, is based on the analysis of 10 units. The AV must remain below the acceptance limit (AV = 15).

AV = |M − X| + ks

M = X, if 98.5 ≤ X ≤ 101.5

M = 98.5, if X < 98.5

M = 101.5, if X > 101.5

K = 2.4

S = standard deviation

#### 3.4.3. Dosing Accuracy and Mass Variation 

Dosing accuracy was expressed as a percentage of dosage units within a batch that meet specified criteria or tolerances for mass variation.

To calculate dosing accuracy, the number of tablets within specification based on mass variation criteria was divided by the total number of tablets, and then multiplied by 100 to express the result as a percentage.

Dosing Accuracy = (the number of tablets within specification/total number of tablets) × 100

The specification for mass variation is derived from the mass uniformity test criteria outlined in *European Pharmacopoeia* (*Ph. Eur.*) 2.9.5. According to these criteria, all tablets underwent mass uniformity evaluation, with quality criteria set at a relative standard deviation (RSD) of 7.5% for 200 mg tablets and 5.0% for 300, 400, and 500 mg tablets.

#### 3.4.4. Dissolution Test 

The dissolution analysis was carried out on a multi-bath (n = 6) dissolution test apparatus 2, 100 rpm for speed (Basket) with dissolution tester DT 128 (Erweka GmbH, Langen, Germany) in accordance with the *United States Pharmacopeia* (*USP*) general methods. All measurements were carried out using a Waters AQUITY ARC HPLC system equipped with a Quaternary solvent manager-R (ACQ-rQSM), a degasser, autosampler (ACQ-rFTN), and photodiode-array detector (2998 PDA). The detector was set at 230 nm.

Samples were withdrawn at specific time intervals of 0, 5, 10, 15, 20, and 30 min. Dissolution was carried out in dilute hydrochloric acid (1 in 100) with a volume of 500 mL. Withdrawn samples underwent filtration through disposable cellulose syringe filters (0.2 µm) to obtain clear solutions. The sinking conditions were maintained by replacing fresh media after each withdrawal from each vessel. These dissolution experiments were conducted during Phases I and III, utilizing MiniLab for Formulation I and Pharma Printer for Formulation II.

#### 3.4.5. Stability Study 

To evaluate the stability of propranolol HCl tablets in blister packing, we conducted a thorough stability study in accordance with ICH Q1 guidelines under long-term conditions (25 ± 2 °C and 60 ± 5% RH) at specific time points (0, 1, 3, and 6 months). This study involved an examination of the formulations at defined intervals throughout the specified duration. Essential parameters, including drug concentration, appearance, and pH were considered when assessing the formulation stability. The analysis was conducted using five tablets for each test to ensure representative sampling and accurate evaluation of stability parameters. The tablets from Formulation I were manufactured during Phase 1, while those from Formulation II were produced during Phase 3. MiniLab was utilized for Formulation I, and Pharma Printer was used for Formulation II.

#### 3.4.6. Statistical Analysis

For our statistical analysis, we utilized a range of methods through SPSS software (version 28, 2021), maintaining a significance level of *p* = 0.05.

## 4. Results and Discussion 

### 4.1. Deposition Accuracy with Mass Variation 

#### 4.1.1. Group I: MiniLab without PS Formulation 

In the study conducted for Group I using MiniLab with Form I in 17 different compounding pharmacy sites across Europe, the focus was on dosing accuracy by assessing mass variation. The experiment involved the alpha testing of MiniLab equipment and the application of Formulation I. The analysis of deposition accuracy was conducted for three different weight categories (500 mg, 400 mg, and 300 mg) across the sites. Table 2 below provides descriptive statistics summarizing the results.

**Figure 5:**
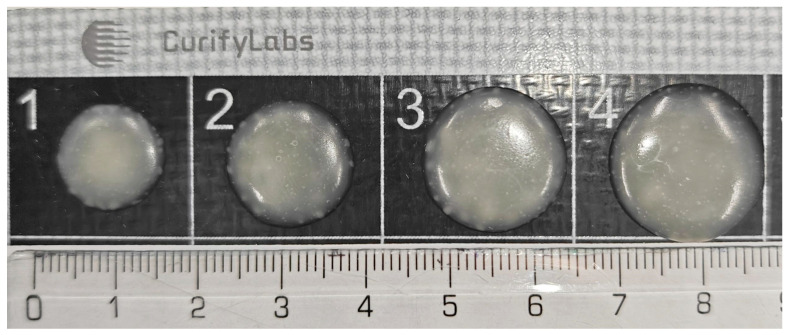
Example of the appearance of the dosed propranolol tablets on a silicon mat: 1. 200 mg; 2. 300 mg; 3. 400 mg; and 4. 500 mg tablets, respectively.

The descriptive statistics include minimum and maximum values, mean, and standard deviation for each weight category. This study indicates that, on average, the dosing accuracy for the 500 mg tablets was 93.8%, demonstrating a relatively high level of accuracy with minimal variation. Similarly, the 400 mg and 300 mg tablets exhibited mean accuracies of 89.2% and 91.2%, respectively. The standard deviations suggest the degree of variability within each weight category. Figure 5 shows a picture of the appearance of the tablets produced in this study. A total of 2448 tablets were made in this phase.

#### 4.1.2. Group II: MiniLab with PS Formulation 

Research was performed for Group II at nine different pharmacies in Europe using MiniLab with Formulation II, again assessing mass variation. This study involved testing MiniLab equipment and using Formulation II. They looked at dosing accuracy for three tablet weights (500 mg, 400 mg, and 300 mg) at each site. Table 3 below summarizes the basic info about dosing accuracy for different tablet weights in Group II. The total number of tablets manufactured was 1296.

These data show the range of values, average accuracy, and variability for each tablet weight. This study found that, on average, the deposition accuracy for 500 mg tablets was 95.8%, indicating a high level of accuracy with little variation. Similarly, the average accuracies for 400 mg and 300 mg tablets were 91.2% and 90.7%, respectively. The variability shows how much the accuracy differs within each tablet weight category.

#### 4.1.3. Group III: Pharma Printer with PS Formulation 

For Group III, the research examined the precision of tablet deposition across various weights: 500 mg, 400 mg, 300 mg, and 200 mg. Here are the key statistics presented in Table 4. The total number of tablets printed in this phase was 2112.

**Figure 6:**
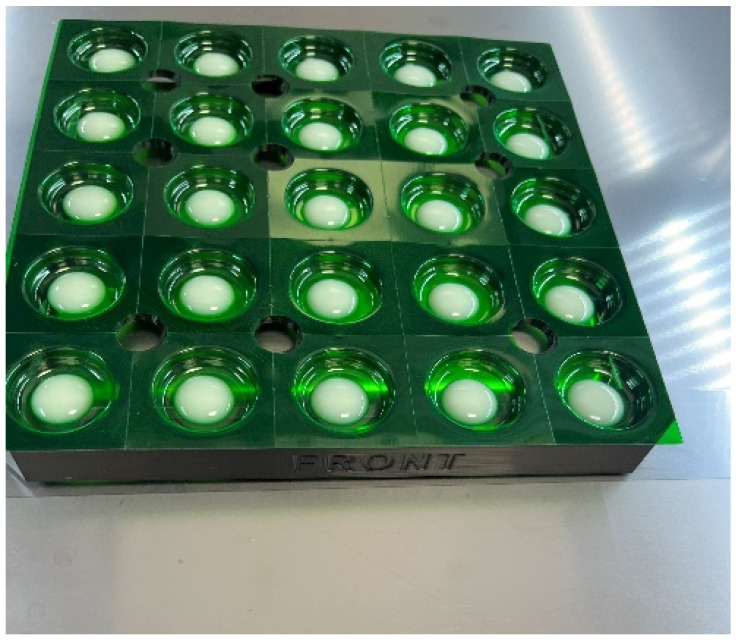
Example of 200 mg tablets dosed into in a 25-blister pack.

The precision of dosing ranged from 93.8% to 100.0%, with mean accuracies between 98.5% and 99.1%. Standard deviations indicate the level of variability in dosing precision within each weight category, which remained relatively low. These results offer valuable insights into the dosing performance of the Pharma Printer with formulation incorporating PS (Form II) across different tablet weights. The observed deposition accuracies highlight the reliability of the equipment across the 11 compounding pharmacy sites, contributing to a comprehensive understanding of its effectiveness in pharmaceutical manufacturing processes. The addition of the 200 mg tablets as a new feature further enhanced the capabilities of the Pharma Printer. In this step, tablets were dosed directly into a blister pack as shown in Figure 6.

The results from Phases 3 and 4 when employing the Pharma Printer indicate a rapid deposition process. The time to deposit a tablet is around 1–3 s per dose including the weight measurement with a scale, which makes it a very rapid approach for compounding and allowing for 100% weight control of each dose. Bendicho-Lavilla [18] reported on a 3D printing approach recently in which in-process weight control was employed. In the study, the most rapid time for one individual tablet printing plus weighing was 50 s for a 250 mg tablet and 75 s for a 750 mg tablet. In comparison, the smallest (200 mg) tablet manufacture with weight control took 1 s with the Pharma Printer, making it 50 times faster than the reported approach. Previously, Lafeber et al. [20] reported on a study with 3D-printed sildenafil and furosemide tablets where the manufacturing method produces tablets with a speed of approximately 1–1.5 tablets per minute, i.e., up to 100 tablets per hour, without any in-process weight control. As a comparison, the 3D printing approaches reported produce roughly 1–1.5 tablets per minute and the automated extrusion-based dosing technology introduced in this study produces 30–60 quality controlled tablets per minute. 

#### 4.1.4. Comparison of Dosing Accuracy across Multiple Tablet Sizes

This study investigated the mean accuracy scores across three different tablet sizes (300 mg, 400 mg, and 500 mg) among three distinct groups. Groups I and II utilized the MiniLab printer, while Group III employed the latest version of the Pharma Printer. The analysis aimed to assess the impact of printer performance on dosing accuracy with mass variation, particularly focusing on dosing accuracy. In total, 3744 tablets were manufactured in the MiniLab group and 2112 tablets in the Pharma Printer group.

**Figure 7:**
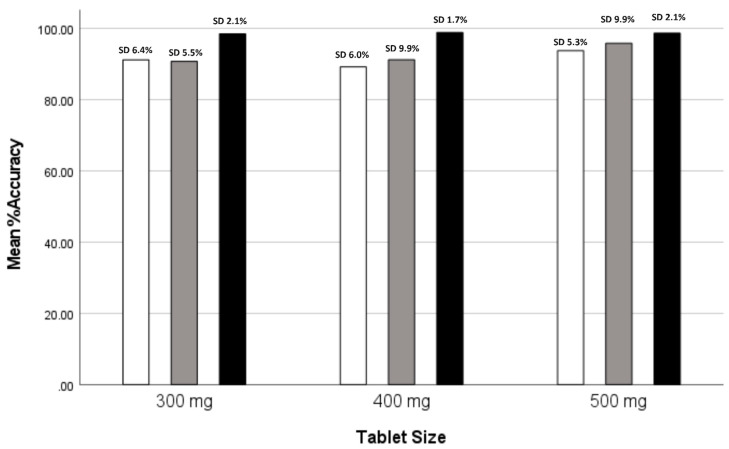
Clustered bar graph: comparison of mean weight accuracy across tablet sizes for three groups: Group I (white), Group II (gray), and Group III (black).

ANOVA results revealed significant differences in mean accuracy scores among the groups for all tablet sizes (Accuracy300: F = 7.838, *p* = 0.002; Accuracy400: F = 7.860, *p* = 0.002; Accuracy500: F = 3.951, *p* = 0.029), suggesting variations in accuracy performance. Robust tests of equality of the mean values further supported these findings, highlighting significant differences in accuracy performance among the groups (*p* < 0.001 for both Accuracy300 and Accuracy400; *p* < 0.001 for Accuracy500). Post-hoc tests indicated that Group III consistently demonstrated significantly higher mean accuracy scores compared to Groups I and II across all tablet sizes. These findings suggest that the utilization of the most advanced version of technology, i.e., the Pharma Printer, in Group III led to superior accuracy performance in dosing compared to the MiniLab printers used by Groups I and II. This study reveals that dosing accuracy with mass variation is indeed dependent on printer performance. No comparison study was conducted for 200 mg tablets as data were available only for Group III using the Pharma printer. Table 5 shows the comparison of mean accuracy scores for three tablet sizes across three groups and Figure 7 shows a comparison of mean weight accuracy across tablet sizes for the three groups.

### 4.2. Content Uniformity Test by HPLC

The comprehensive analysis of content uniformity (CU) demonstrates consistent drug content across the varied tablet sizes. Each tablet size, from 200 mg to 500 mg, exhibits a narrow distribution of drug content, as evidenced by the low relative standard deviation (RSD%) values. This uniformity is crucial to ensure that each tablet delivers the intended dosage. The acceptance value (AV), a critical parameter indicating content uniformity, is well within acceptable limits for all tablet sizes. The mean values, reflecting the average drug content, and SD values, indicative of variability, are also within established parameters, underscoring the uniformity of the formulation.

The content uniformity analysis establishes that the 1% propranolol HCl with 1% polysorbate in CuraBlend^®^ tablets maintain uniform composition across different tablet sizes. The consistently low RSD%, AV, mean, and SD values affirm the precision and reliability of the manufacturing process. This outcome reinforces the confidence in the quality and uniform drug delivery capabilities of the tablets, contributing to the overall quality and safety of the pharmaceutical product. 

#### 4.2.1. Group I: MiniLab with Formulation I

For the content uniformity (%CU) analysis, each tablet size underwent testing with a sample size of 170. The %CU values varied, with the minimum and maximum percentages falling within acceptable ranges: 79.0% to 115.9% for 500 mg tablets, 79.5% to 118.1% for 400 mg tablets, and 84.6% to 111.9% for 300 mg tablets. The mean %CU values were 102.9% for 500 mg tablets, 103.5% for 400 mg tablets, and 103.1% for 300 mg tablets(Table 6) While the mean values for all tablet sizes are within the acceptance criteria, the standard deviations (7.7, 6.7, and 6.9, respectively) suggest some variability in content uniformity.

Upon closer examination of the data, it becomes evident that despite the mean values falling within the acceptable range, there are individual %CU values that deviate slightly from the specified limits. This deviation could be attributed to various factors within the formulation and manufacturing process, including homogeneity issues during mixing.

One potential explanation for these deviations could be the absence of polysorbate during the mixing of the active pharmaceutical ingredient (API) in advance and its subsequent transfer to CuraBlend^®^. This absence may have resulted in inadequate homogenization of the mixture, leading to the presence of some outliers in the content uniformity analysis.

Therefore, it is imperative to consider the influence of formulation and manufacturing process variables on content uniformity outcomes. Addressing issues related to homogeneity in the formulation process could help mitigate the occurrence of outliers and ensure consistent and reliable content uniformity results.

#### 4.2.2. Group II: MiniLab with Formulation II

The data illustrate CU results for tablet sizes of 500 mg, 400 mg, and 300 mg, with additional insight into the AV measurements (Table 7). Notably, these formulations incorporated polysorbate to enhance the mixing of the API with the formulation, aimed at improving CU results.

Across all tablet sizes, the mean %CU values align closely with the acceptable range of 85.0% to 115.0%. Specifically, the mean %CU values are as follows: 103.4% for 500 mg tablets, 103.4% for 400 mg tablets, and 103.1% for 300 mg tablets. The standard deviations for %CU are relatively low, ranging from approximately 3.9 to 4.9, indicating consistent content uniformity results within each tablet size group.

Furthermore, the AV data provide insight into the variability within the CU test. For the 500 mg tablets, the AV ranges from 2.0 to 17.0, with a mean value of 11.4 and a standard deviation of 5.4. Similarly, for the 400 mg tablets, the AV ranges from 6.0 to 15.0, with a mean value of 10.8 and a standard deviation of 3.3. Additionally, for the 300 mg tablets, the AV ranges from 8.0 to 19.0, with a mean value of 13.5 and a standard deviation of 3.2. These findings suggest variability in content uniformity results across different tablet sizes, potentially influenced by the limitations in MiniLab’s dosing accuracy. Moreover, MiniLab’s dosing accuracy outliers affected the entire batch, impacting even the minimum, maximum, and mean values of CU. Although these parameters fall within the acceptable range, the presence of outliers widened the range of results and consequently affected the AV values. Notably, two outlier AV values were observed, highlighting the need for improvement in deposition accuracy.

Addressing the dosing accuracy issues in MiniLab lead to enhanced content uniformity and reduced variability across different tablet sizes. By improving deposition accuracy, the consistency and reliability of content uniformity outcomes was significantly enhanced. These aspects were taken into account in the improvements made for the Pharma Printer that was used in Phase 3 and Phase 4 of this study.

#### 4.2.3. Group III: Pharma Printer with Formulation II

The data for Group III presents content uniformity (CU) results for tablet sizes of 500 mg, 400 mg, 300 mg, and 200 mg, along with corresponding acceptance value (AV) measurements (Table 8). It is important to note that the formulations for Group III are consistent with those of Group II, utilizing a Pharma Printer for production.

Across all tablet sizes, the mean %CU values fall within the acceptable range of 85.0% to 115.0%. Specifically, the mean %CU values are as follows: 103.2% for 500 mg tablets, 103.5% for 400 mg tablets, 103.2% for 300 mg tablets, and 104.3% for 200 mg tablets. The standard deviations for %CU vary slightly, ranging from approximately 3.4 to 3.9, indicating consistent content uniformity results within each tablet size group.

**Figure 8:**
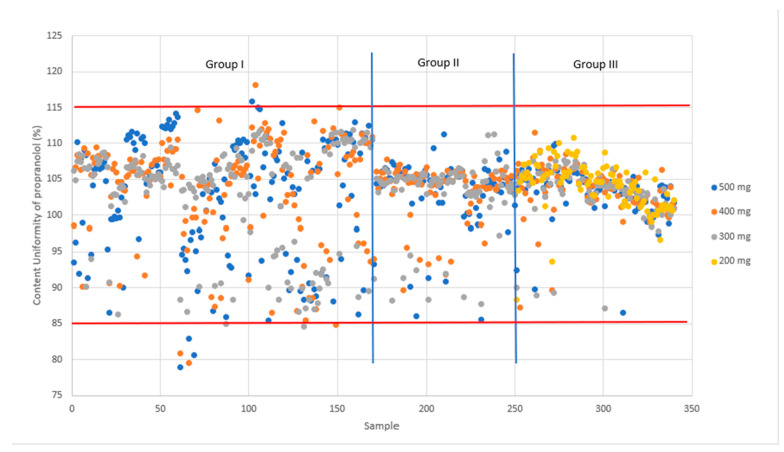
Content uniformity of propranolol without PS80 (Group I) and with PS80 (Groups II and III) analyzed from 34 batches (1110 tablets) and all tablet sizes (200–500 mg). Groups I and II were printed using NM printer and Group III using Pharma Printer. Acceptance limits are shown in red. Blue lines are separators for the different groups.

Additionally, the AV data provide insight into the variability within the CU test. For the 500 mg tablets, the AV ranges from 3.0 to 14.0, with a mean value of 8.2 and a standard deviation of 4.2. Similarly, for the 400 mg tablets, the AV ranges from 3.0 to 16.0, with a mean value of 8.6 and a standard deviation of 5.0. Furthermore, for the 300 mg tablets, the AV ranges from 3.00 to 16.0, with a mean value of 9.2 and a standard deviation of 5.6. Finally, for the 200 mg tablets, the AV ranges from 4.0 to 15.0, with a mean value of 8.8 and a standard deviation of 4.2. Figure 8 clearly shows the tighter CU values as this study progresses from Phase 1 to Phase 3. The spread in the results is much larger in Phase 1 and 2 compared Phase 3.

#### 4.2.4. Comparison of Acceptance Value of Content Uniformity Test across Multiple Tablet Sizes: Between Group I, II, and III

This study investigated the mean acceptance value (AV) across three distinct tablet sizes (300 mg, 400 mg, and 500 mg) among three groups, each employing different formulations and printers (Table 9). Group I utilized formulations without polysorbate and the MiniLab printer, while Groups II and III employed formulations with polysorbate, with Group III additionally utilizing the latest Pharma Printer. The analysis aimed to discern the impact of polysorbate inclusion in formulations and printer type on AV with content uniformity, specifically focusing on AV of content uniformity test.

ANOVA outcomes unveiled noteworthy variances in mean AV among the groups for all tablet sizes (AV300: F = 5.483, *p* = 0.009; AV400: F = 11.223, *p* < 0.001; AV500: F = 6.834, *p* = 0.003), indicating differences in AV performance. Robust tests of equality of means further validated these findings, underscoring significant disparities in AV performance among the groups (*p* = 0.024 for AV300, *p* = 0.001 for AV400, and *p* = 0.004 for AV500).

Post-hoc tests revealed that Group III consistently exhibited notably lower mean AV compared to Groups I and II across all tablet sizes. These findings suggest that the utilization of polysorbate-containing formulations and the latest Pharma Printer in Group III led to superior AV performance compared to the MiniLab printers used by Groups I and II with similar formulations. This highlights the combined influence of formulation composition and printer performance on AV measurements with content uniformity, with the Pharma Printer demonstrating enhanced accuracy in dosing.

This study underscores the interplay of both polysorbate inclusion in formulations and printer type on AV with content uniformity. The utilization of polysorbate-containing formulations and the Pharma Printer in Group III resulted in superior AV performance compared to Groups I and II. These findings underscore the importance of optimizing both formulation composition and printer type for improved accuracy in dosing pharmaceutical products.

#### 4.2.5. Group IV: Pharma Printer with Hardware Updates and Formulation II

The data from Group IV as shown in Table 10 showcase exceptional precision in dosing across different tablet sizes. This part of the study was conducted in the R&D laboratory of CurifyLabs. The Pharma Printer was employed with a more rigid syringe holder and dosing weight increments of 25 mg were introduced. The testing protocol with 3x 16 tablets for 200, 300, 400, and 500 mg target weights was tested, and dosing was also demonstrated with target weights of 225, 275, and 425 mg, respectively, which are three randomly chosen target weights in between the fixed values in the protocol. All in all, 336 tablets were dosed in this phase. The mean values for mass variation (MV) demonstrate consistent accuracy, with minimal deviations observed for all. The accuracy as defined per *Ph. Eur.* limits for the tablets in all target weight classes was 100%. For the 225 mg tablets, the mass variation ranges from 218.0 to 230.0, with a mean of 224.5 and a standard deviation of 2.2. Similarly, for the 275 mg tablets, the mass variation remains tightly clustered, ranging from 264.0 to 281.0, with a mean of 274.0 mg and a standard deviation of 3.1. The 425 mg tablets also exhibit precision in dosing, with values concentrated around the mean of 423.6 and a standard deviation of 4.3. 

These findings highlight the effectiveness of the Pharma Printer’s new syringe attachment and dose in ensuring precise control of the dosing. The narrow ranges and minimal deviations in mass variation across all tablet sizes indicate that the printer can accurately dispense doses within the specified ranges. It is worth noting that this test and the selection of tablet weights were based on a challenge test aimed at checking the printer’s performance with specific tablet weights, such as 225 mg, 275 mg, and 425 mg. This level of accuracy is crucial in pharmaceutical manufacturing to guarantee that patients receive the intended dosage of medication consistently. 

**Figure 9:**
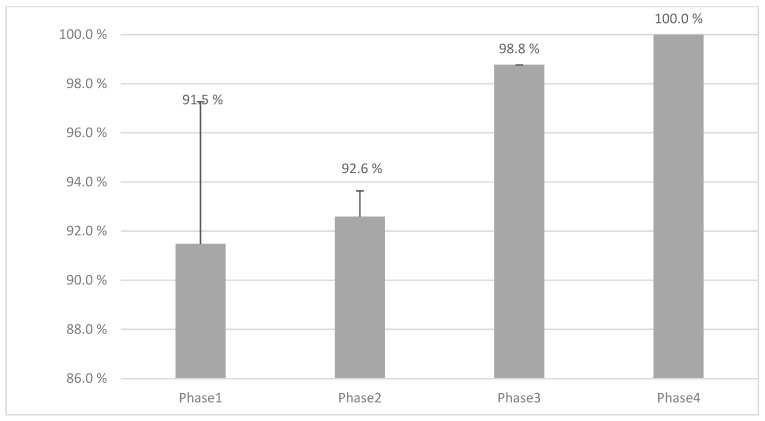
The mean weight accuracy (%) observed across the four phases of this study. In Phase 1, a total of 2448 tablets were used. Phase 2 utilized 1152 tablets, while Phase 3 involved 2112 tablets. Phase 4, which marks the final step, utilized a total of 336 tablets. It is noteworthy that Phases 1 and 2 were conducted using the MiniLab, while Phases 3 and 4 utilized the Pharma Printer extrusion-based dispenser. Remarkably, the figure demonstrates 100% accuracy achieved in the final step, reflecting the efficacy of the implemented improvements.

Figure 9 shows the dosing accuracy (%) and standard deviations across all phases of this study showing 100% accuracy in the final step when all improvements have been implemented. Phase 1 and 2 were performed with the MiniLab and Phases 3 and 4 with the Pharma Printer, indicating a high dependency of the hardware features on the accuracy dosing weight.

The acceptance value, a critical parameter in pharmaceutical analysis, denoting the uniformity of dosage units, was calculated to be 2. Coupled with a standard deviation of 0.85, these metrics offer a comprehensive understanding of the variability and consistency within the tablet batch. This finding underscores the robustness and precision of the manufacturing process, particularly with the implementation of hardware updates in the Pharma Printer. It reflects a diligent adherence to quality control measures, ensuring that each tablet within the batch delivers the intended dose with reliability and accuracy (Table 11). 

### 4.3. Blend Uniformity Test by NIR Spectroscopy

**Figure 10:**
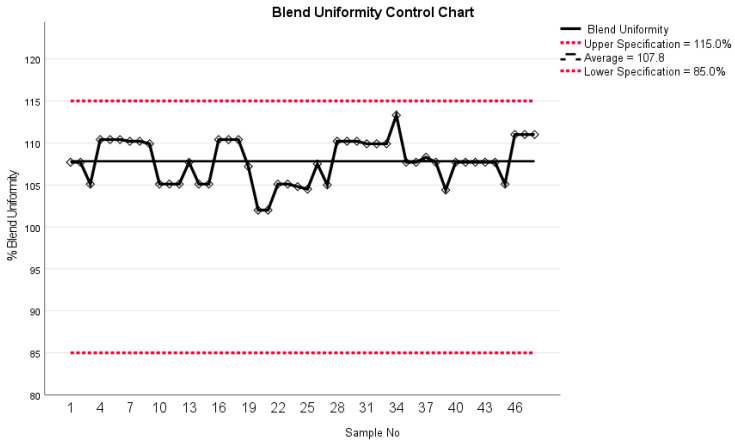
Blend uniformity control chart from NIR spectroscopy.

In earlier studies, NIR spectroscopy was used, for instance, for quantitative measurement of accurate dosing of theophylline [21], levothyroxine, and prednisolone using inkjet printing [22]. Recently Seani-Viano et al. [23] reported on a case study of 3D-printed efavirenz tablets and successful real-time NIR measurements of them. Blend uniformity results indicated a mean of 107.8% with a standard deviation of 2.6% (Figure 10). These data fall within the specified range of 90 to 110%, aligning with the internal specification for blend uniformity. Thus, they meet the acceptance criteria for content uniformity, which typically ranges from 85% to 115%.

However, it is important to note that some values slightly exceed the upper limit of 110%. This may be attributed to inadequate mixing during the mixing process, resulting in localized areas with higher concentrations of the active ingredient. While these outliers are minimal, they underscore the significance of thorough blending to ensure homogeneity across the entire batch. Furthermore, the ongoing improvement in the partial least squares (PLS) model for blend uniformity remains a continuous process. This involves refining the model-building process to enhance its accuracy and predictive capabilities. The CU and AV values form the HPLC analyses confirm the high quality of blend uniformity. It is important to understand that the NIR analysis utilizes a very small sample amount, approximately 25–50 mg of sample.

### 4.4. Dissolution Test 

**Figure 11:**
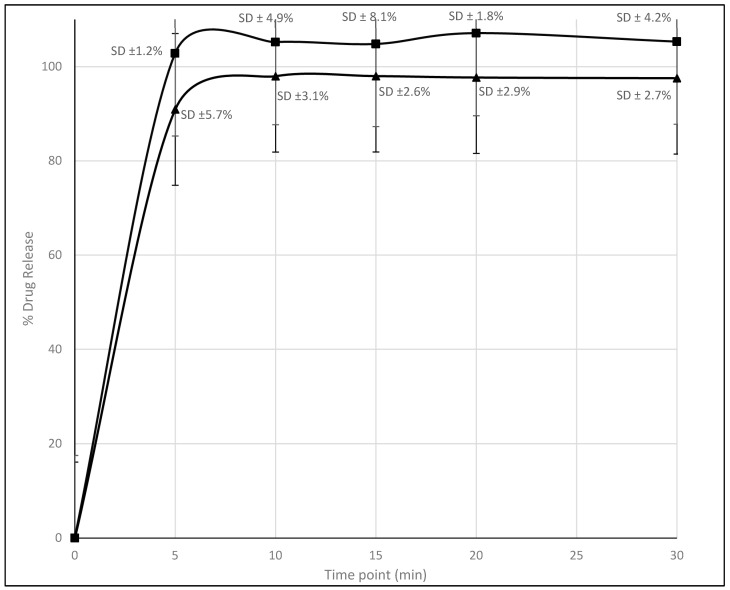
Dissolution profiles: Formulation I vs. Formulation II with error bars (▲ for Formulation I and ■ for Formulation II).

The Figure 11 illustrates that both formulations primarily release the majority of the drug within the initial 5 min. However, Formulation II demonstrates a slightly higher drug release, surpassing 100%, potentially attributed to overestimation or experimental error, as indicated by fluctuations above 100%. Conversely, Formulation I exhibits a release that maintains at approximately 98% from the 5 min mark onwards. The presence of polysorbate in Formulation II likely contributes to the slightly improved dissolution characteristics, resulting in a sharper drug release profile initially. This observation suggests that the addition of polysorbate has the potential to enhance the dissolution behavior of the formulation, as reported regarding the ability of polysorbate to enhance solubility and dissolution rates of poorly water-soluble drugs [24].

### 4.5. Stability Study 

In the stability study, the appearance of the tablets from both Formulation I and Formulation II was meticulously evaluated over the initial three-month period (Table 12). The tablets maintained their anticipated off-white, soft, chewable form with a vanilla flavor, confirming adherence to the specified characteristics. This visual conformity underscores the stability of the formulations in preserving their physical attributes throughout the assessment’s duration.

Assessing the drug potency through the assay results revealed consistent performance for both formulations. Formulation II exhibited values of 105.5% at zero months, 107.6% at the first month, and 105.7% at the third month. The observed values within the assessed period consistently fell within the acceptance range of 90.0–110.0%. This indicates that both formulations maintain their specified potency limits, ensuring the sustained efficacy of the medication.

Drawing conclusions from the stability data, it can be asserted that the 1% propranolol HCl with 1% polysorbate in CuraBlend^®^ tablets, represented by both Formulation I and Formulation II, exhibits stability for at least three months under the recommended storage conditions (room temperature). The confirmation of appearance conformity and consistent drug potency over time provides a robust foundation for the formulations’ reliability and potential for long-term use.

Examining the specific attributes of each formulation, Formulation II showcased conformity in appearance throughout the three-month period, with pH values consistently within the range of between 4.9 and 5.0. In contrast, Formulation I maintained its appearance and exhibited pH values within the specified range of between 4.5 and 5.5. However, the assay results for Formulation I indicated a gradual decrease in drug potency, with values declining from 95.3% at zero months to 92.3% at the third month. Table 11 provides a concise overview of the stability study results for both Formulation I and Formulation II, encompassing appearance, pH values, and drug potency at zero, first, and third months.

### 4.6. Overall Summary of the Results

This study explored the accuracy of tablet deposition and content uniformity across four groups, employing different printers and formulations, to assess the impact of technological advancements and formulation modifications on pharmaceutical manufacturing precision.

#### 4.6.1. Deposition Accuracy with Mass Variation

Group I (MiniLab with Formulation I) demonstrated deposition accuracies ranging from 89.2% to 93.8% across tablet weights of 300 mg to 500 mg, suggesting a high level of accuracy with minimal variation.

- Group II (MiniLab with Formulation II) showed improved accuracies, particularly for 500 mg tablets at 95.8%, indicating the positive impact of Formulation II on printing precision.
- Group III (Pharma Printer with Formulation II) achieved superior accuracies between 98.5% and 99.1%, highlighting the effectiveness of advanced dosing technology combined with Formulation II.
- Group IV introduced hardware updates to the Pharma Printer and tested additional target weights (225 mg, 275 mg, 425 mg), achieving 100% accuracy within the *European Pharmacopoeia* limits, showcasing exceptional precision in dosing across various tablet sizes.

#### 4.6.2. Content Uniformity

- Consistent drug content was maintained across all tablet sizes in Groups I to III, with Group III displaying slightly better uniformity and lower variability in acceptance value (AV) than Groups I and II.
- Group IV data focused on deposition accuracy, indicating a shift towards optimizing hardware for enhanced precision, and demonstrated very accurate content uniformity and low AV values.

#### 4.6.3. Blend Uniformity and Dissolution Tests

- Blend uniformity met the internal specification for content uniformity, indicating the successful mixing of the active ingredient throughout the batches.
- Dissolution tests for two formulations highlighted differences in drug release profiles, suggesting formulation-dependent effects on dissolution kinetics.

#### 4.6.4. Stability Study

- Over a three-month period, both formulations maintained their physical attributes and drug potency within the accepted range, affirming the stability and efficacy of the medication.

#### 4.6.5. General Considerations

For streamlining processes using automated technology for compounding purposes, there is a need to have ready-made excipient bases available that can be utilized in a versatile way in extemporaneous drug manufacturing. If pharmacies need to develop these themselves, a considerable amount of resources need to be put into this, and this is not typically possible in compounding pharmacies or hospital pharmacies due to lack of resources. It is of course essential that these excipient bases or pharma-inks are of the highest quality and are GMP-manufactured. Moreover, the suitability for pediatric use needs to be assured. Van Kampen et al. [25] wrote brief overview of carrier materials currently used in pharmaceutical extrusion-based printing studies of medicines for pediatrics and they elaborated on how to guide in carrier material selection in this type of application [26,27,28,29]. Many studies have addressed the importance of pediatric considerations, and the STEP (Safety and Toxicity of Excipients for Pediatrics) database [30] and FDA’s equivalent database [31] are relevant sources in the context.

As we delve deeper into the digital transformation of manufacturing, leveraging cutting-edge technologies like artificial intelligence (AI) and the Internet of Things (IoT) becomes increasingly central. These advancements are paving the way for highly personalized and automated production methods across both public and private sectors. Within this landscape, three-dimensional (3D) printing emerges as an intriguing innovation, especially in crafting complex, tailored products with new functionalities [32,33,34,35,36,37,38].

The application of 3D printing in the pharmaceutical sector, specifically in drug compounding, can replace current manual methods when speed, accuracy, quality control, and user-friendliness are fulfilled. While research into personalized pharmaceuticals has been extensive, aiming to transform traditional manufacturing processes, the technology’s role in personalizing drug delivery systems is not without challenges. The concept of tailoring medication to individual patient needs through 3D printing—adjusting drug shapes, sizes, and dosages, or adding specific release functionalities—remains complex and not fully realized. The practicality of implementing such personalized production on a wider scale, especially for standard medication compounding, reveals the technology’s constraints in terms of efficiency and adaptability.

However, while 3D printing offers remarkable possibilities for creating tailored drugs for individual needs, it is an efficient solution only when speed and ease of use are addressed. Straightforward automated extrusion-based material deposition technologies and GMP-manufactured excipient bases as presented in this multi-site study offer a more viable alternative for rapid automated compounding. These methods are precise and excel in speed, and the simplification of quality control processes, making them better suited for the fast-paced production of standard pharmaceuticals that are needed in normal compounding scenarios where the produced dosage forms should be similar in function (bioequivalent) to market-authorized products. This distinction underscores the need to choose the right manufacturing technology based on the specific requirements of drug production, whether seeking customization and complexity or efficiency, standardization, and scalability in drug compounding.

Acknowledging this study’s limitations is integral to ensuring transparency and guiding future research endeavors. While our focus on propranolol HCl allowed for an in-depth exploration of the technology’s application, this study’s generalizability to other APIs is viable. We have extensive feasibility and quality control data for many other drugs from all Biopharmaceutical Classification System (BCS) classes, such as furosemide. Furosemide falls under BCS Class IV, characterized by low permeability and low solubility. 

## 5. Conclusions

This study underscores the critical role of both printer technology and formulation enhancements in achieving high levels of precision and uniformity in pharmaceutical manufacturing. The introduction of hardware updates in the last phase of this study, alongside specific target weight testing, marked a significant advancement in dosing accuracy, achieving 100% compliance with pharmacopeial standards. These findings highlight the importance of continuous technological and formulation improvements to ensure accurate, uniform, and stable pharmaceutical products. The results and insights gained from this extensive multinational study pave the way for the future of pharmacy-tailored personalized medicine by modernizing manual compounding practices and improving patient outcomes.

This multi-site study concerning the automation of oral pediatric tablet compounding using a novel technology inspired by 3D printing with in-process quality control tools was made between May 2023 and December 2023, and in total, 30 hospital and community pharmacies from eight European countries took part in it. The results indicate that extemporaneous pharmacy manufacturing can take a giant leap forward towards automation and digital manufacture of dosage forms through this automated and quality controlled compounding approach. This type of technology has become reality in pharmacies, because it is able to cater to a wide range of drug substances and dosage forms and presents an alternative to laborious manual extemporaneous manufacturing, thanks to validated and standardized steps in the process. Moreover, the methods used need to be fast and validated to make sense for a pharmacy to invest in technology to replace manual compounding techniques. Several 3D printing approaches that have been proposed in the past years, are very slow and their use typically requires a lot of technical competence for operating [9].

Pharmacy compounding stands at the forefront of transformation, not least due to demands of closed-loop medication management requirements by unit dose labelling and traceability due to a digital process. Traditional methods of compounding often involve manual and time-consuming processes, presenting challenges in terms of consistency, dosage accuracy, and scalability. However, the emergence of cutting-edge technologies has started a new era for pharmacy compounding, promising to redefine the way medications are prepared and delivered. Looking forward, continued innovation and collaborokation within the industries and pharmacies will be essential to further refine automated compounding systems, optimize processes for efficiency and scalability, and realize the full potential of personalized medicine.

## Figures and Tables

**Table 1:** HPLC gradient conditions for propranolol HCl analysis.

Time (min)	Flow Rate (mL/min)	% Buffer	% Acetonitrile
0.00	0.700	80.0	20.0
2.00	0.700	80.0	20.0
7.00	0.700	20.0	80.0
8.00	0.700	80.0	20.0
12.00	0.700	80.0	20.0

**Table 2:** Descriptive statistics of average deposition accuracy across different tablet weights in group I.

Tablet Weights	N	Minimum (%)	Maximum (%)	Mean (%)	Std. Deviation
500 mg	17	83.3	100.0	93.8	5.3
400 mg	17	79.2	97.9	89.2	6.0
300 mg	17	79.2	100.0	91.2	6.4

In Table 2, “N” represents the number of printing sites. For each weight test performed, three sets of 16 tablets were printed, resulting in a total of 48 tablets printed for each weight in each size. Therefore, the total number of printed tablets analyzed across all sites is 816 tablets per weight category.

**Table 3:** Descriptive statistics of average dosing accuracy across different tablet weights in group II.

Tablet Weights	N	Minimum (%)	Maximum (%)	Mean (%)	Std. Deviation
500 mg	9	81.3	100.0	95.8	5.9
400 mg	9	72.9	97.9	91.2	9.9
300 mg	9	81.3	97.9	90.7	5.5

In Table 3, “N” represents the number of printing sites. With three sets of 16 tablets printed for each weight test, a total of 48 tablets were produced for each weight in every size. Hence, the cumulative number of tablets scrutinized across all sites is 432 per weight category.

**Table 4:** Descriptive statistics of average deposition accuracy across different tablet weights in Group III.

Tablet Weight	N	Minimum (%)	Maximum (%)	Mean (%)	Std. Deviation
500 mg	11	93.8	100.0	98.5	2.1
400 mg	11	95.8	100.0	98.9	1.7
300 mg	11	93.8	100.0	98.5	2.1
200 mg	11	95.8	100.0	99.1	1.4

The number of printing sites involved is shown by “N” in Table 4. For every weight test, three sets of 16 tablets were printed; this resulted in a total of 48 tablets overall, 1 for each weight and size. As a result, 528 tablets per weight category were analyzed in total across every site.

**Table 5:** Comparison of mean accuracy scores for tablet sizes (300 mg, 400 mg, and 500 mg) across three groups.

Tablet Size	Comparison	Mean Difference	Standard Error	*p*-Value	95% Confidence Interval
300 mg	Group I vs. Group II	0.43425	2.16209	0.980	[−5.0999, 5.9684]
	Group I vs. Group III	7.30898 *	2.02951	0.004	[2.1142, 12.5038]
	Group II vs. Group III	7.74323 *	2.35738	0.009	[1.7092, 13.7773]
400 mg	Group I vs. Group II	−1.98797	2.63780	0.755	[−8.7398, 4.7639]
	Group I vs. Group III	9.64717 *	2.47605	0.002	[3.3094, 15.9850]
	Group II vs. Group III	7.65919 *	2.87606	0.040	[0.2975, 15.0209]
500 mg	Group I vs. Group II	−2.54497	2.53453	0.608	[−3.9425, 9.0325]
	Group I vs. Group III	4.92513	2.37911	0.133	[−1.1645, 11.0148]
	Group II vs. Group III	−7.47010 *	2.76346	0.037	[−14.5436, −0.3966] *

* Significant at the 0.05 level.

**Table 6:** Content uniformity (CU) and acceptance value (AV) analysis for different tablet sizes—Group I (MiniLab without PS formulation).

Variable	N	Minimum (%)	Maximum (%)	Mean (%)	Std. Deviation
%CU 500 mg	170	79.0	115.9	102.9	7.7
%CU 400 mg	170	79.5	118.1	103.5	6.7
%CU 300 mg	170	84.6	111.9	103.1	6.9
AV 500 mg	17	2.0	27.0	16.5	6.2
AV 400 mg	17	9.0	26.0	16.8	4.7
AV 300 mg	17	5.0	26.0	16.6	6.1

In Table 6, “N” represents the number of tablets that were analyzed for each variable. The acceptance value data show variability within the content uniformity (CU) test across different tablet sizes. Across all tablet sizes (500 mg, 400 mg, and 300 mg), the mean AV values are relatively consistent, ranging from approximately 16.47 to 16.76. However, the ranges of AV values vary within each tablet size group, indicating some variability in content uniformity results.

**Table 7:** Content uniformity (CU) and acceptance value (AV) analysis for different tablet sizes—Group II (MiniLab with PS formulation).

Variable	N	Minimum (%)	Maximum (%)	Mean (%)	Std. Deviation
%CU 500 mg	80	85.6	111.3	103.4	4.7
%CU 400 mg	80	89.7	107.8	103.4	3.9
%CU 300 mg	80	87.8	111.3	103.1	4.9
AV 500 mg	8	2.0	17.0	11.4	5.4
AV 400 mg	8	6.0	15.0	10.8	3.3
AV 300 mg	8	8.0	19.0	13.5	3.2

The number of tablets that were examined for each variable is indicated by the letter “N” in Table 7.

**Table 8:** Content uniformity (CU) and acceptance value (AV) analysis for different tablet sizes—Group III (Pharma Printer with Formulation II). “N” in Table 8 indicates the total number of tablets analyzed in each variable.

Variable	N	Minimum (%)	Maximum (%)	Mean (%)	Std. Deviation
%CU 500 mg	90	86.5	109.9	103.2	3.4
%CU 400 mg	90	87.3	111.5	103.5	3.4
%CU 300 mg	90	87.1	108.7	103.2	3.8
%CU 200 mg	90	88.4	110.8	104.3	3.4
AV 500 mg	9	3.0	14.0	8.2	4.2
AV 400 mg	9	3.0	16.0	8.6	5.0
AV 300 mg	9	3.0	16.0	9.2	5.6
AV 200 mg	9	4.0	15.0	8.8	4.2

**Table 9:** Comparison of mean acceptance value (AV) for tablet sizes (300 mg, 400 mg, and 500 mg) across three groups.

Tablet Size	Comparison	Mean Difference (Group I vs. Group II)	Mean Difference (Group I vs. Group III)	Mean Difference (Group II vs. Group III)	Standard Error	*p*-Value (Group I vs. Group II)	*p*-Value (Group I vs. Group III)	*p*-Value (Group II vs. Group III)	Significant at 0.05 Level
300 mg	Group I vs. Group II	2.2	6.2	4.2	1.4	0.016	0.001	0.065	Yes
	Group I vs. Group III	7.3	-	5.2	1.2	0.003	-	0.029	Yes
	Group II vs. Group III	5.1	-	-	1.8	0.022	-	-	No
400 mg	Group I vs. Group II	5.0	7.9	2.9	1.6	0.081	0.003	0.159	No
	Group I vs. Group III	9.0	-	3.0	1.5	0.001	-	0.067	Yes
	Group II vs. Group III	4.0	-	-	1.8	0.037	-	-	No
500 mg	Group I vs. Group II	4.0	7.8	3.8	1.6	0.039	0.004	0.041	Yes
	Group I vs. Group III	7.8	-	3.8	1.5	0.010	-	0.043	Yes
	Group II vs. Group III	3.8	-	-	1.7	0.044	-	-	Yes

No comparison study was conducted for 200 mg tablets, as data were only available for Group III utilizing the Pharma Printer.

**Table 10:** Tablet weights using Pharma Printer with new syringe attachment and dose slider.

Tablet Weight	Number of Tablets	Minimum (mg)	Maximum (mg)	Mean (mg)	Std. Deviation
200 mg	48	193	208	200.5	3.2
300 mg	48	289	314	300.8	5.0
400 mg	48	382	420	401.8	6.4
500 mg	48	485	521	502.0	6.7
225 mg	48	218.0	230.0	224.5	2.2
275 mg	48	264.0	281.0	274.0	3.1
425 mg	48	408.0	437.0	423.6	4.4

**Table 11:** Content uniformity test results for 10 tablets manufactured with Pharma Printer hardware updates and Formulation II.

Content Uniformity%	Tablet Number
100.2	1
100.9	2
101.4	3
102.1	4
100.4	5
102.4	6
101.4	7
102.7	8
100.9	9
101.1	10
101.3	**Average (%)**
0.85	**Standard deviation**
2	**Acceptance value**

**Table 12:** Stability report for tablets from Formulation I and Formulation II. An overview of the stability study results for both Formulation I and Formulation II, encompassing appearance, pH values, and drug potency at zero, first, and third months. For each test, five tablets were used to ensure representative sampling and accurate evaluation of stability parameters over the specified time intervals.

Test	Specification	Zero Month	First Month	Third Month	Six Month
**Formulation I—Phase 1**					
Appearance	Off-white, soft, chewable tablet with vanilla flavor	Conforms	Conforms	Conforms	Conforms
pH	4.5–5.5	5.0	5.0	5.0	5.1
Assay	90.0–110.0%	95.3%	95.2%	92.3%	94.1%
**Formulation II—Phase 3**					
Appearance	Off-white, soft, chewable tablet with vanilla flavor	Conforms	Conforms	Conforms	Conforms
pH	4.5–5.5	4.9	4.9	5.0	5.0
Assay	90.0–110.0%	105.5%	107.6%	105.7%	107.6%

## Data Availability

The original contributions presented in the study are included in the article and further inquiries can be directed to the corresponding author.
